# A204E mutation in Na_v_1.4 DIS3 exerts gain- and loss-of-function effects that lead to periodic paralysis combining hyper- with hypo-kalaemic signs

**DOI:** 10.1038/s41598-018-34750-8

**Published:** 2018-11-12

**Authors:** Yosuke Kokunai, Carine Dalle, Savine Vicart, Damien Sternberg, Valérie Pouliot, Said Bendahhou, Emmanuel Fournier, Mohamed Chahine, Bertrand Fontaine, Sophie Nicole

**Affiliations:** 1 Inserm U1127, CNRS UMR 7225, Sorbonne Universités, UPMC Univ Paris 06 UMR S 1127, Institut du Cerveau et de la Moelle épinière (ICM), F-75013 Paris, France; 2 AP-HP, Hôpital Universitaire Pitié-Salpétrière, National Reference Center for Channelopathies, F-75013 Paris, France; 3 Centre de recherche CERVO, Institut Universitaire en Santé Mentale de Québec, Quebec City, QC G1J 2G3 Canada; 4 Department of Medicine, Université Laval, Quebec City, QC G1K 7P4 Canada; 5 CNRS UMR7370, LP2M, Labex ICST, University Nice Sophia-Antipolis, Faculté de Médecine, Nice, France

## Abstract

Periodic paralyses (PP) are characterized by episodic muscle weakness and are classified into the distinct hyperkalaemic (hyperPP) and hypokalaemic (hypoPP) forms. The dominantly-inherited form of hyperPP is caused by overactivity of Na_v_1.4 — the skeletal muscle voltage-gated sodium channel. Familial hypoPP results from a leaking gating pore current induced by dominant mutations in Na_v_1.4 or Ca_v_1.1, the skeletal muscle voltage-gated calcium channel. Here, we report an individual with clinical signs of hyperPP and hypokalaemic episodes of muscle paralysis who was heterozygous for the novel p.Ala204Glu (A204E) substitution located in one region of Na_v_1.4 poor in disease-related variations. A204E induced a significant decrease of sodium current density, increased the window current, enhanced fast and slow inactivation of Na_v_1.4, and did not cause gating pore current in functional analyses. Interestingly, the negative impact of A204E on Na_v_1.4 activation was strengthened in low concentration of extracellular K^+^. Our data prove the existence of a phenotype combining signs of hyperPP and hypoPP due to dominant Na_v_1.4 mutations. The hyperPP component would result from gain-of-function effects on Na_v_1.4 and the hypokalemic episodes of paralysis from loss-of-function effects strengthened by low K^+^. Our data argue for a non-negligible role of Na_v_1.4 loss-of-function in familial hypoPP.

## Introduction

**Figure 1:**
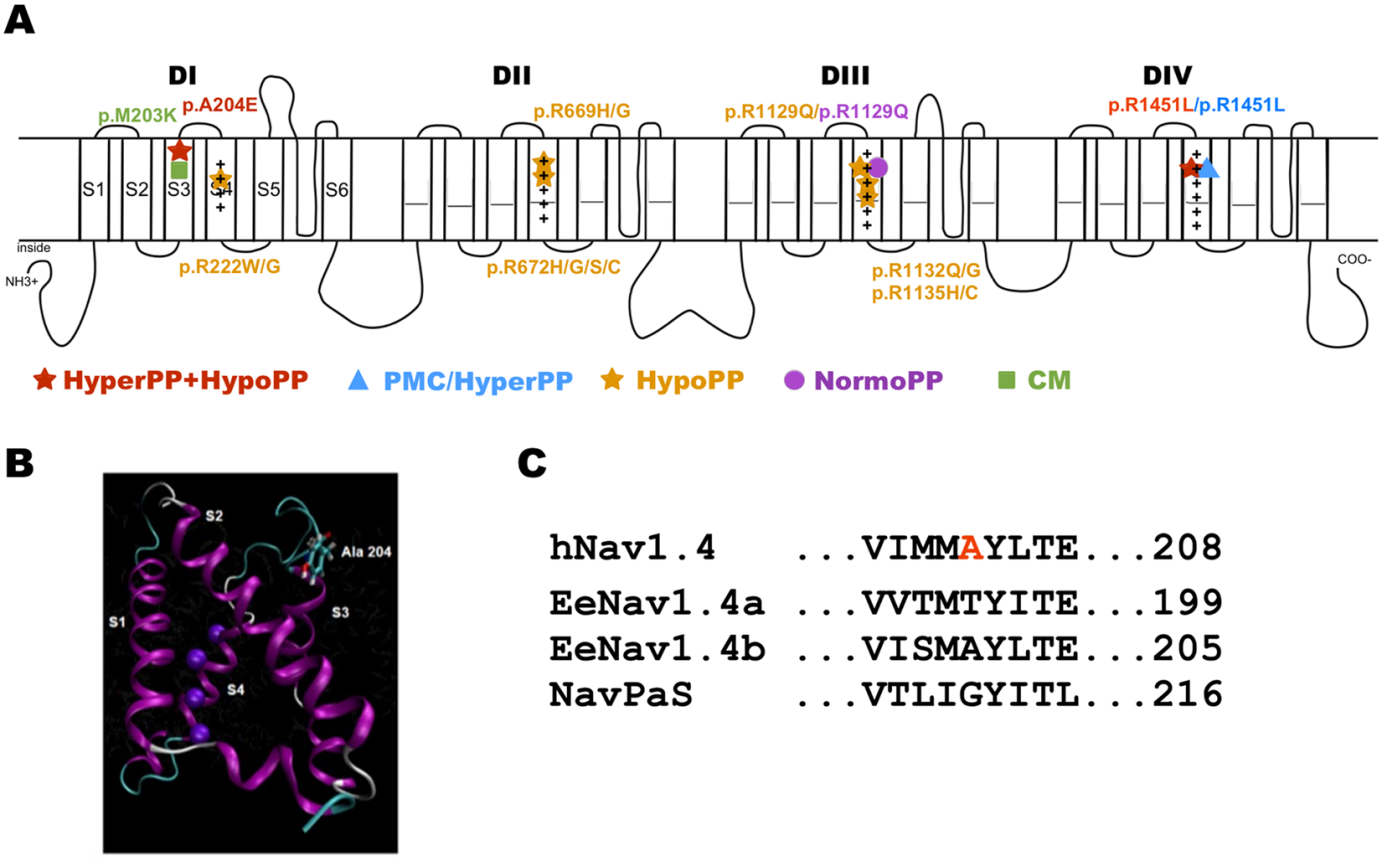
Schema of the pore-forming α subunit of Na_v_1.4 and location of A204E. (**A**) The pore-forming α subunit of hNa_v_1.4 is composed of 1.836 amino acid residues forming 4 homologous domains (DI-DIV). Each domain is composed of 6 transmembrane segments (S1–S6). The S1–S4 segments of each domain form the voltage-sensor domain, with the positively-charged S4 segments acting as voltage-sensors while the S5 and S6 segments form the selective α pore. More than 70 mutations have been described in human Na_v_1.4. Only the ones located in DIS3 or phenotypically-related to A204E are listed on the schema: p.M203K located in DIS3 and associated to a congenital myopathy phenotype; the hypoPP missense mutations substituting positively-charged (+) residues in S4 segments; p.R1451L resulting in a PP phenotype combining hyper- and hypo-PP. The p.R1129Q and p.R1451L are associated with two distinct phenotypes: hypoPP or normoPP (R1129Q), and *paramyotonia congenita* with hyperPP or PP combining hyper- and hypo-PP signs (R1451L). Hyper PP = hyperkalaemic periodic paralysis; hypoPP = hypokalaemic periodic paralysis; PMC = *paramyotonia congenita*; NormoPP = normokalaemic PP; CM = congenital myopathy. (**B**) Model of voltage-sensor domain I based on Na_v_Ab crystal structure^40^ showing the position of the Ala204 residue on the extracellular side of S3. (**C**) Alignment of the hNa_v_1.4 amino acid sequence around the A204 residue with eukaryotic EeNav1.4 (electric eel) and Na_V_PaS (putative Na_v_ channel from the American cockroach) channels whose cryo-electron microscopy structure was determined with a high-level resolution. The A204 residue is not conserved in these orthologs except in EeNav1.4b.

Periodic paralyses (PP) are characterized by episodic muscle weakness classified into hypokalaemic (hypoPP), hyperkalaemic (hyperPP), familial and non-familial forms^1^. Non-familial forms of PP are frequently secondary to another physiological dysfunction such as thyrotoxicosis, hyperaldosteronism or nephropathic K^+^ loss for hypoPP, and chronic kidney disease of K^+^ supplements for hyperPP. Familial forms of PP are dominantly-inherited^2^. Most of them result from missense mutations in the *SCN4A* gene that encodes the pore forming α subunit of Na_v_1.4, the skeletal muscle voltage-gated Na^+^ channel. Na_v_1.4 belongs to the family of voltage-gated sodium channels (VGSC). Na_v_1.4 is composed of one pore-forming α subunit and one auxiliary β subunit. The pore-forming α subunit is a monomer that folds into four homologous but not-identical domains (DI-DIV). Each domain is composed of six transmembrane α helices called segments (S1–S6, Fig. 1A). Segments 1 to 4 form the Voltage Sensor Domain (VSD) that undergoes conformational shifts in response to membrane potential depolarization, thereby driving the opening of the central pore domain formed by the S5–S6 segments^3^. The conformational changes in response to depolarization are driven by the outward movement of repetitively occurring positively charged residues (also called gating charges) in S4, which is facilitated by electrostatic interactions of these basic (mostly Arg but also Lys) residues with conserved acidic or polar residues in S2 and S3 segments. The linker between DIII and DIV and its Ile/Phe/Met motif are key elements for fast inactivation.

Six skeletal muscle channelopathies in human result from *SCN4A* mutations and form a clinical spectrum ranging from muscle hyperexcitability (myotonia due to delayed muscle relaxation) to hypoexcitability (muscle weakness resulting in fetal hypokinesia for the most severe form)^4^. PP are in between the extreme forms, although secondary permanent muscle weakness may develop independently from the paralytic attacks with aging (up to 68% of patients over 41 years)^5^. All Na_v_1.4 channelopathies have an autosomal dominant mode of inheritance except those with primary congenital muscle weakness (myasthenia, myopathy and hypokinesia), which result from recessively-inherited loss-of-function mutations of *SCN4A*. Dominant *SCN4A* mutations resulting in hyperPP usually impair the inactivation properties of Na_v_1.4^4,6^. Dominant *SCN4A* mutations resulting in hypoPP substitute one positively charged Arg residue located in a S4 segment of DI, II or III (Fig. 1A). Familial HypoPP mutations induce a gating pore current at the resting state, which is an inward cationic current through a non-selective aqueous pathway created by the neutralization of one of the two most extracellular positive charges of S4 segments^7–9^. The gating pore current results in paradoxical depolarization of the sarcolemma in low extracellular K^+^, which is supposed to be at the origin of episodic muscle paralysis^2,10^.

Determining whether PP is hypo- or hyper-kalaemic is clinically important since K^+^ intake improves hypoPP and worsens hyperPP. We recently reported one individual who suffered from an unusual phenotype of PP combining hyperkalaemic symptoms with hypokalaemic episodes of muscle paralysis and was heterozygous for a *SCN4A* mutation encoding the p.Arg1451Leu (p.R1451L) substitution located in DIVS4^11^. Here, we report a second individual with a similar phenotype, who was also found heterozygous for a novel (p.Ala204Glu) substitution in Na_v_1.4, and the biophysical changes induced by this mutation on Na_v_1.4 current to gain insight the molecular mechanisms leading to this unusual form of congenital PP.

## Results

### Clinical investigations

The proband is a 39-year-old Caucasian male without any reported familial history of neuromuscular disease. He is the second of three male infants born from non-consanguineous parents. He first experienced painful cramps in lower limb muscles at low temperatures at the age of 12: he complained from muscle discomfort with cramps and muscle pain that lasted less than one hour. He then reported muscle stiffness of jaw and hands at cold, suggesting cold-aggravated myotonia during all his adolescence. At the age of 16, he started to suffer from quadriplegia at wake up with a frequency of one per one or two months, usually when resting after intensive exercise or taking carbohydrates-rich food at diner. Hypokalaemia (around 2 mM) was confirmed several times during these attacks, and the patient was successfully treated with K^+^ uptakes. Thereafter, paralytic attacks changed both in frequency and symptoms with a combination of short and long paralytic attacks. The short crises occurred several times in the day and lasted less than one hour. The long episodes lasted for 2–3 hours, included quadriplegia and occurred with a frequency of 3–4 times per month. At the age of 31, their frequency increased up to one per day with duration up to 48 hours. A provocative test consisting in a night meal rich in carbohydrates (pizza) induced a long attack with concomitant decreased level of blood K^+^ at 2.3 mM, again indicative of hypoPP. Acetazolamide with K^+^ supplement (600 mg per day) was then introduced and was found to reduce the occurrence of long paralytic attacks. At last examination, the patient did not complain from any muscle deficit between attacks, and no clinical signs arguing for secondary permanent muscle weakness were observed.

Laboratory examinations were normal, excluding thyrotoxicosis or renal dysfunction. Intercritical dosages of CPK done at several occasions were slightly increased (300–350 UI/l at the age of 33 years). Needle EMG examination done at the ages of 26 and 32 detected myotonic discharges at room temperature. Functional EMG showed gradual increment of compound muscle action potentials (CMAP) amplitudes after repeated brief exercise tests (effect of cold not tested) and decremental CMAP amplitudes in response to long exercise (equivalent to McManis) test, which was compatible with Fournier’s classification type 4 that we most frequently observed in hyperPP^12,13^.

### Heterozygous mutation in DIS3 of Na_v_1.4

*SCN4A*, *CACNA1S*, *CLCN1* and *KCNJ2* were screened for mutations by sequencing of peripheral blood DNA. No candidate mutation was found in *CACNA1S*, *CLCN1* or *KCNJ2*, whereas a novel missense substitution (c.611 C > A) was identified at the heterozygous state in exon 4 of *SCN4A*. This transversion encodes the p.Ala204Glu amino acid substitution (thereafter referred as p.A204E). The A204 residue would be located close to the extracellular side of the third segment (S3) of domain I (DIS3, Fig. 1A,B). The A204E substitution was not observed in our cohort of individuals suffering from skeletal muscle channelopathies nor reported in single nucleotide polymorphism database. In the latter, the p.Ala204Val substitution (c.611 C > T, rs199859508) was observed in 4 alleles from African population on 35.190 chromosomes (ExAc database). The Ala204 amino acid residue is invariant among human Na_v_ paralogs and Na_v_1.4 orthologs of mammals but is not conserved in the two eukaryotic Na_v_1.4 channels with 4 Å molecular structure resolved by cryo-electron microscopy (Fig. 1C and data not shown)^14,15^. The replacement of the non-polar Ala residue by the negatively charged Glu residue in position 204 was classified as disease-causing by three algorithms used for pathogenicity prediction (Polyphen, Sift and Mutation Taster).

### Biophysical properties of the A204E mutant channel

**Figure 2:**
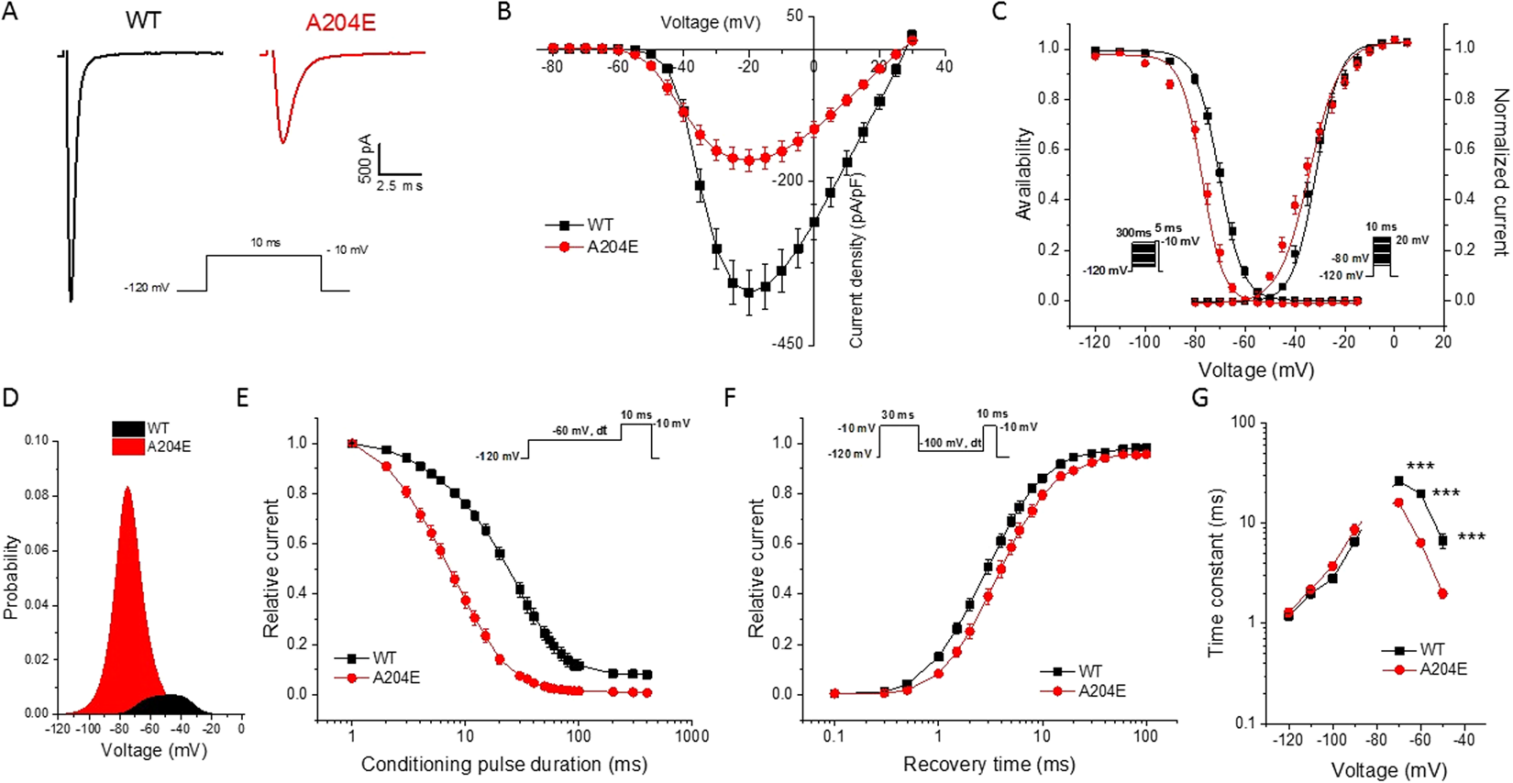
Biophysical properties of wild-type (WT) and mutant A204E hNa_v_1.4 channels in HEK293 cells. (**A**) Representative whole-cell current traces recorded from HEK293 cells expressing wild-type (WT, black) or A204E (red) hNa_v_1.4 channels in response to a test pulse of 10 ms at −10 mV from a holding potential of −120 mV are shown. (**B**) Normalized current/voltage relationships of WT (*n* = 36) and A204E (*n* = 28) channels. Current densities were normalized by cell capacitance. (**C**) Voltage dependence of activation and steady-state fast inactivation curves for WT and A204E channels were plotted and fitted with a single Boltzmann equation. (**D**) Effect of A204E on window current. The probability of being within this window was calculated as indicated in the materials and methods section. (**E**) Steady-state fast inactivation for WT and A204E channels in response to the pulse protocol shown in the inset. **(F**) Recovery from fast inactivation of WT and A204E channels. (**G**) Voltage-dependence of time constants (τ) of fast inactivation for WT (*n* = 9) and A204E (*n* = 9) channels obtained when measuring entry from −70 to −30 mV and recovery from −120 to −80 mV. The statistical differences between WT and A204E channels are shown (***P < 0.001).

We first studied the voltage-dependence of activation, steady-state fast inactivation and slow inactivation in HEK293 cells transiently transfected with WT or A204E human Na_v_1.4 channels using the whole-cell configuration of the patch-clamp technique. Figure 2A displays representative traces of voltage-dependent Na^+^ currents obtained from cells expressing WT or A204E channels. The current density of A204E channels was reduced by 2-fold compared to WT channels (Fig. 2A,B, WT: 393 ± 34 pA/pF, A204E: 174 ± 17 pA/pF, ***P < 0.001). The A204E mutation caused hyperpolarizing shifts of mid-point values (V_1/2_) of voltage-dependence curves of steady state activation (*V*_1/2_, WT: −32.0 ± 1.1 mV, A204E: −35.0 ± 1.1 mV, *P < 0.05) and fast-inactivation (*V*_1/2_, WT: −69.7 ± 0.7 mV, A204E: −76.0 ± 0.7 mV, ***P < 0.001) compared with WT channel (Fig. 2C and Table 1). The slope factor of the activation curve was increased for A204E compared to WT (Fig. 2C and Table 1, *k*_*v*_, WT: 3.9 ± 0.2, A204E: 6.4 ± 0.2, ***P < 0.001). By contrast, it was decreased for steady-state inactivation (Fig. 2C and Table 1, *k*_*v*_, WT: 4.0 ± 0.0, A204E: 3.7 ± 0.1, ***P < 0.001). All these changes predicted that the probability of being within the window current — a range of voltages where the probability of Na_v_1.4 opening is significant — was increased by 7-fold for A204E compared to WT with a hyperpolarizing shift of −18 mV for the highest probability as shown in Fig. 2D.

**Table 1:** Voltage dependence of steady-state activation, fast and slow inactivation for WT and mutant A204E hNa_v_1.4 channels.

	Steady state activation		Fast inactivation		Slow inactivation	
	*V*_1/2_ (mV)	*k*_*v*_	*V*_1/2_ (mV)	*k*_v_	*V*_1/2_ (mV)	*k*_v_
WT	−32.0 ± 1.1 (36)	3.9 ± 0.2 (36)	−69.7 ± 0.7 (36)	4.0 ± 0.0 (36)	−69.5 ± 0.9 (11)	5.0 ± 0.2 (11)
A204E	−35.0 ± 1.1 (28)*	6.4 ± 0.2 (28)***	−76.0 ± 0.7 (28)***	3.7 ± 0.1 (28)***	−77.0 ± 0.8 (14)**	5.1 ± 0.2 (14)

V_1/2_, midpoint for activation or inactivation (mV); k, slope factor for activation or inactivation. The numbers of cells (n) are in brackets. Values are means ± sem. *P < 0.05, **P < 0.01, ***P < 0.001.

The kinetics of entry into fast inactivation and of recovery from fast inactivation were further evaluated using a three-pulse protocol as shown in Fig. 2E,F. The entry into fast inactivation of A204E channels was accelerated compared to WT channels whereas the kinetics of recovery did not differ significantly (Fig. 2G). Precisely at voltages higher than −70 mV, the time constants (τ) of entry into fast inactivation for the A204E channels were smaller than the WT time constants (Fig. 2G, τ, WT: 26.5 ± 2.6 ms, A204E: 16.0 ± 1.2 ms at −70 mV, ***P < 0.001; WT: 19.5 ± 1.4 ms, A204E: 6.3 ± 0.4 ms at −60 mV, ***P < 0.001; WT: 6.7 ± 1.0 ms, A204E: 1.9 ± 0.2 ms at −50 mV ***P < 0.001; *n* = 9 for each condition).

**Figure 3:**
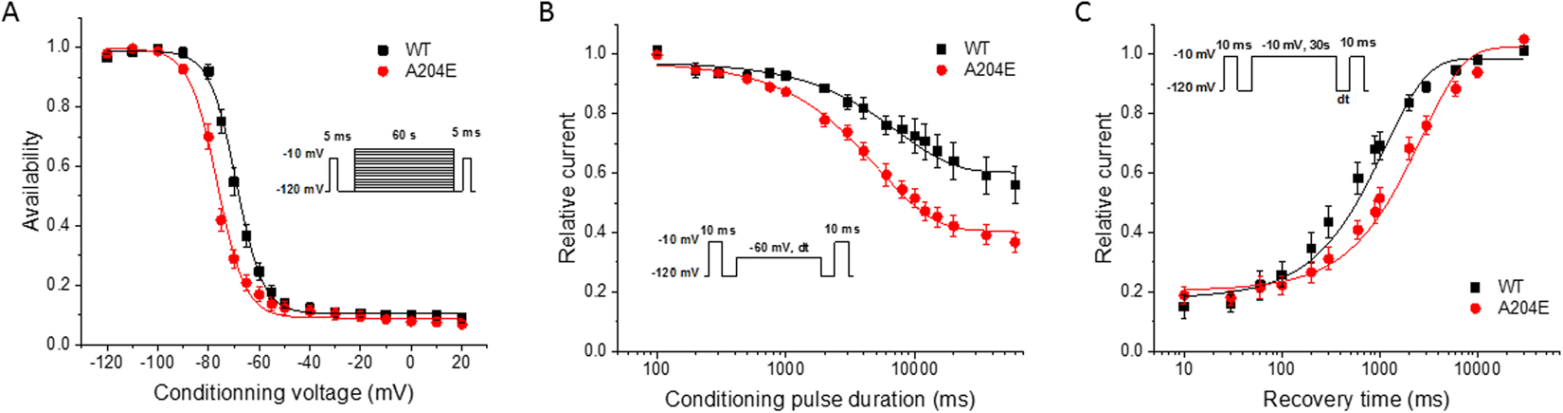
Analyses of slow inactivation of wild-type (WT) and mutant (A204E) hNa_v_1.4 channels in HEK293 cells. (**A**) The voltage dependence of entry into slow inactivation was measured using a triple-pulse protocol. The cells were first depolarized at −10 mV for 5 ms, then for 60 sec from a holding potential at −120 mV to +20 mV. A 5 ms test pulse at −10 mV was applied to measure the Na^+^ current after an interval of 20 ms at −120 mV to allow recovery from fast inactivation. (**B**) The entry into slow inactivation was also measured with a triple-pulse protocol. The resulting curves were fitted to a monoexponential equation. (**C**) Recovery from slow inactivation. The resulting curves were fitted with a monoexponential equation.

Slow inactivation was assessed using triple-pulse protocols (Fig. 3). The voltage-dependence of slow inactivation for A204E channels was shifted by 7.5 mV toward hyperpolarization compared to WT channels (*V*_1/2_, WT: −69.5 ± 0.9 mV, A204E: −77.0 ± 0.8 mV, **P < 0.01, Fig. 3A and Table 1). The entry of A204E channels into the slow inactivated state was dramatically accelerated compared to WT channels (τ, WT: 8145.5 ± 1701.2 ms, A204E: 4242.8 ± 337.3 ms at −60 mV, *P < 0.05, Fig. 3B). On the contrary, the recovery from slow inactivation was slowed down by A204E (τ, WT: 627.5 ± 100.2 ms, A204E: 1540.8 ± 178.9 ms at −100 mV, ***P < 0.001; Fig. 3C).

### Gating pore current analysis

**Figure 4:**
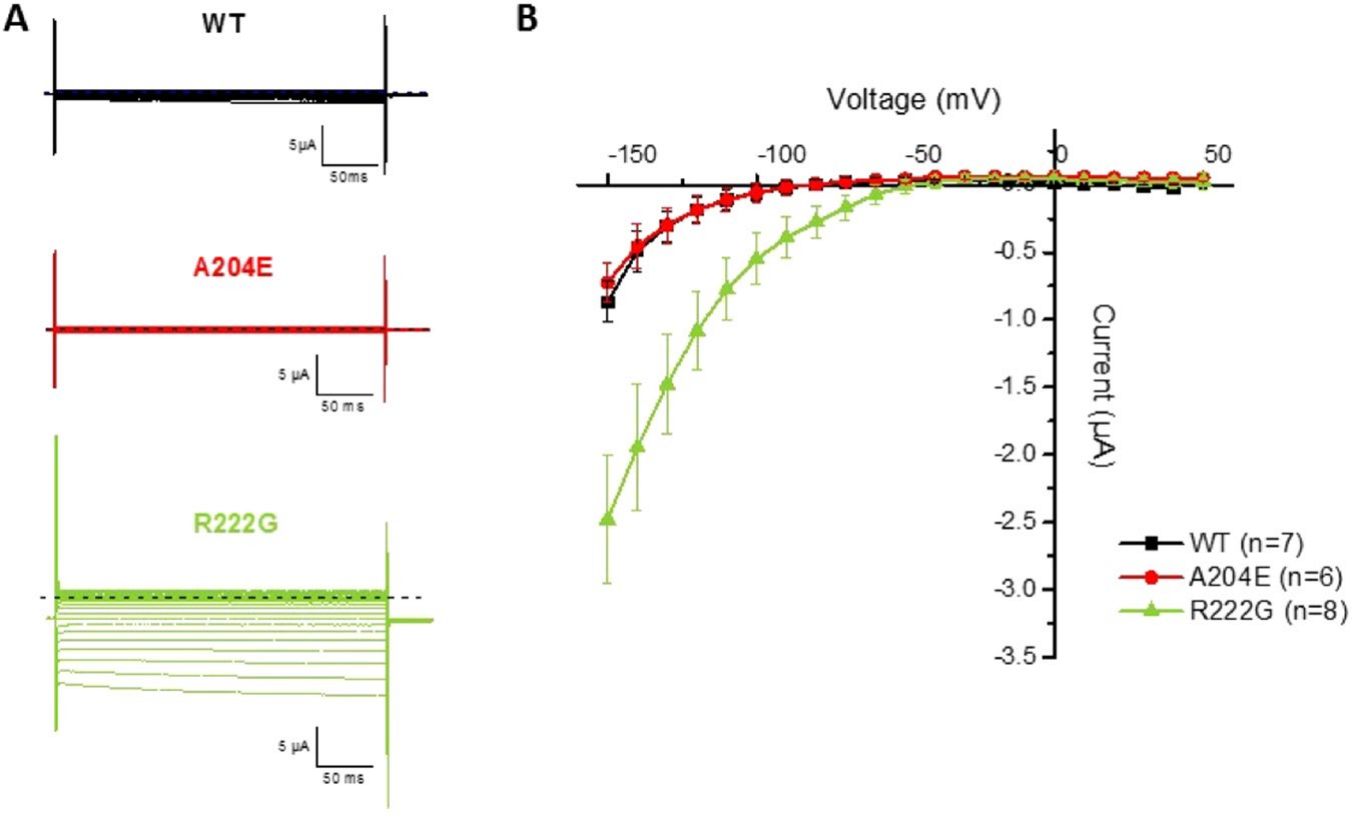
Analyses of gating pore current in oocytes. (**A**) Representative current traces recorded in response to stimulation protocols from oocytes expressing WT (top, in black), A204E (middle, in red) or R222G (bottom, in green) rNa_v_1.4 channels. The dashed line in black represents the zero current. (**B**) Normalized current-voltage relationships (I-V) for WT, A204E and R222G rNa_v_1.4 channels.

The dominant Na_v_1.4 mutations resulting in familial hypoPP are now well-known to cause a gating pore current, and we evaluated the possibility that A204E generates such a current *in vitro*^16–18^. The p.Arg222Gly (p.R222G) substitution — a hypoPP mutation located in DI known to induce gating pore current — was used as a positive control^10^. Oocytes were held at −80 mV and those with maximum α currents larger than 20 µA were perfused with 1 µM tetrodotoxin to block the α pore. Current recording protocol consisted of 300 ms voltage pulses from −150 mV to +20 mV in 10 mV increments while maintaining the oocyte at a holding potential of −80 mV. An offline subtraction of the linear leak (−50 to +50 mV) was used and extrapolated to the raw data. The mean current during the last 50 ms of the test pulse was plotted against the voltage to analyze the current-voltage relationship (I-V) of the gating pore current. As shown in Fig. 4, no gating pore current was generated by p.A204E in contrast to p.R222G.

### K^+^ sensitivity of mutant A204E channels

**Figure 5:**
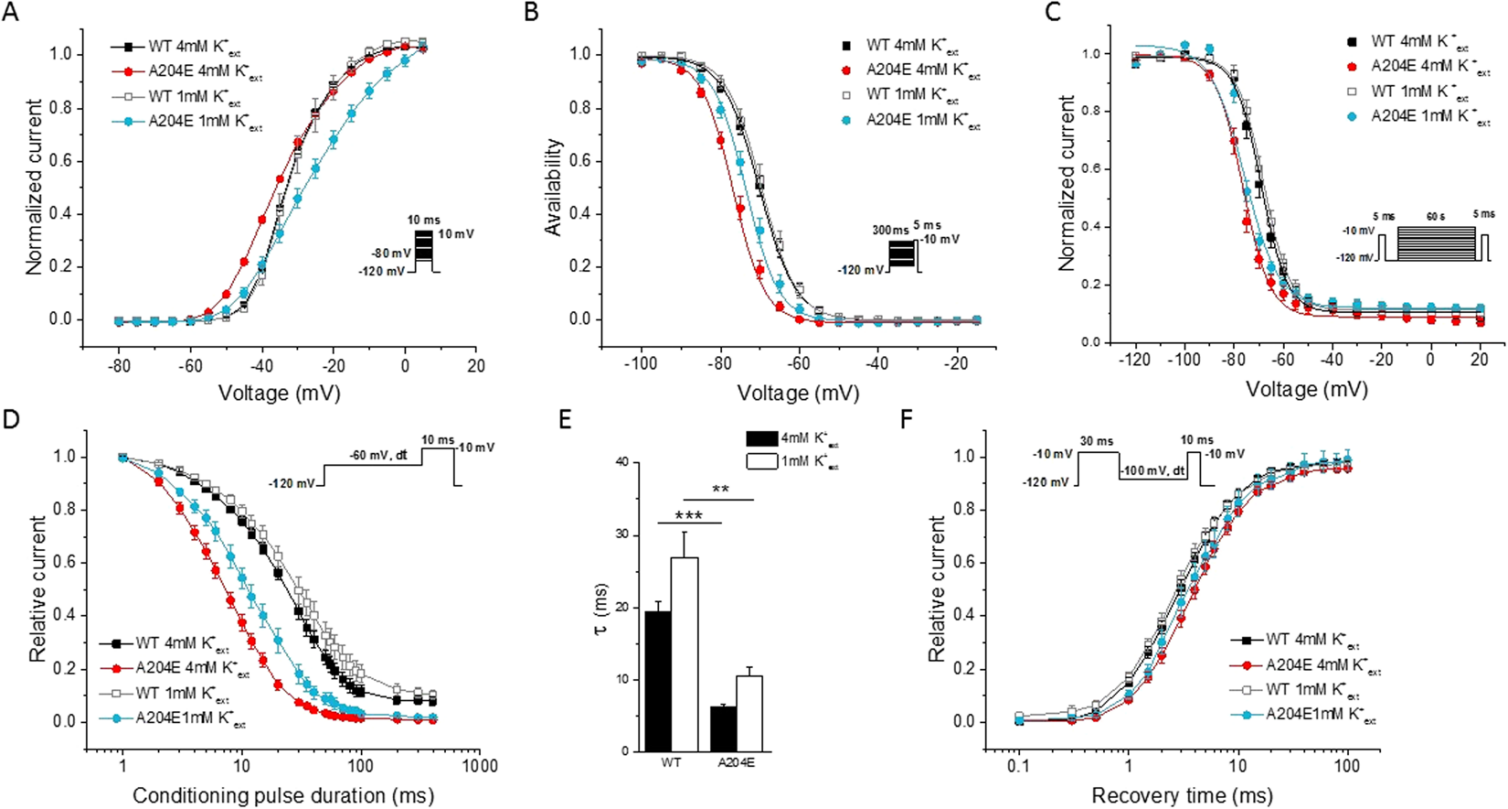
Biophysical properties of WT and mutant hNa_v_1.4 channels in an extracellular concentration of K^+^ equal to 1mM (1 mM K^+^_ext_) compared to 4 mM K^+^_ext_. (**A**) Voltage-dependence of steady-state activation. (**B**) Voltage-dependence of steady-state fast inactivation. (**C**) Voltage-dependence of slow inactivation. (**D**) Entry into fast inactivation. (**E**) Time constants of entry into fast inactivation at −60 mV. The statistical differences between WT and A204E values at 4 mM K^+^_ext_ and 1 mM K^+^_ext_ channels are shown (**P < 0.01; ***P < 0.001). (**F**) Recovery from fast inactivation obtained with a holding potential of −100 mV.

Sensitivity of Na_v_1.4 gating behavior to the variation of extracellular K^+^ concentration was reported for some *SCN4A* mutations such as p.Gly1306Ala/Glu/Val (p.G1306A/E/V), which are located in the cytoplasmic DIII-DIV linker and cause K^+^-aggravated myotonia^19^. We therefore tested the effect of low extracellular K^+^ concentration on A204E channels. Na^+^ current density for A204E channels compared to WT channels was similarly reduced at 1 mM and 4 mM K^+^_ext_ (data not shown). The voltage-dependence of steady-state activation and fast inactivation of WT and A204E channels at 1 mM and 4 mM K^+^_ext_ were compared (Fig. 5 and Table 2). In our experimental conditions, the WT channel gating behavior was insensitive to low K^+^_ext_ concentration. By contrast, some biophysical properties of A204E channels were modified when reducing K^+^_ext_. The most important changes were observed for steady-state activation. Its voltage-dependence was shifted by 4.5 mV toward depolarization compared to WT channels at 1 mM K^+^, whereas it was shifted toward hyperpolarization by 3 mV at 4 mM K^+^_ext_ (Fig. 5A and Table 2, *V*_1/2_, A204E at 4 mM K^+^_ext_: −35.0 ± 1.1 mV, A204E at 1 mM K^+^_ext_: −27.4 ± 1.4 mV, ***P < 0.001). The activation curve of A204E channels was also less steep at 1 mM K^+^_ext_ compared to 4 mM K^+^_ext_, thereby amplifying the difference with WT channels (Fig. 5A and Table 2, *k*_*v*_, A204E at 4 mM K^+^_ext_: 6.4 ± 0.2; A204E at 1 mM K^+^_ext_: 7.9 ± 0.2, **P < 0.01). On the contrary, the differences observed for the voltage-dependence of fast inactivation between A204E and WT channels at 4 mM K^+^_ext_ were attenuated at 1 mM K^+^_ext_ (Fig. 5B and Table 2, *V*_1/2_, WT at 1 mM K^+^_ext_: −69.4 ± 1.1 mV, A204E at 1 mM K^+^_ext_: −72.9 ± 0.9 mV, **P < 0.01). The change of slow inactivation properties induced by p.A204E were not affected when decreasing K^+^_ext_ (Fig. 5C and data not shown). Low K^+^_ext_ slightly modified the kinetics of entry into fast inactivation at −60 mV for both the WT and A204E channels (Fig. 5D). The reduction of τ value for entry into fast inactivation observed for A204E channels compared to WT channels was nevertheless still observed at 1 mM (Fig. 5D,E, τ, WT-1 mM K^+^_ext_: 26.9 ± 3.6 ms, A204E-1 mM K^+^_ext_: 10.5 ± 1.2 ms, **P < 0.01). No significant change was observed for the recovery from fast inactivation (Fig. 5F; τ, WT-1 mM K^+^_ext_: 2.8 ± 0.3 ms, A204E-1 mM K^+^_ext_: 3.5 ± 0.5 ms, *P* = 0.2). Therefore, low ${{\rm{K}}}_{{\rm{ext}}}^{+}$ disfavored the activated state and still favored the slow and fast inactivation states for A204E channels compared to WT channels.

**Table 2:** Voltage dependence of steady-state activation, fast and slow inactivation in 4 and 1 mM extracellular K^+^ concentrations.

	Activation		Fast inactivation		Slow inactivation	
	*V*_1/2_ (mV)	*k*_*v*_	*V*_1/2_ (mV)	*k*_*v*_	*V*_1/2_ (mV)	*k*_*v*_
**WT**						
4 mM K^+^	−32.0 ± 1.1 (36)	3.9 ± 0.2 (36)	−69.8 ± 0.7 (36)	4.0 ± 0.0 (36)	−69.5 ± 0.9 (11)	5.0 ± 0.2 (11)
1 mM K^+^	−31.9 ± 1.6 (14)	4.0 ± 0.2 (14)	−69.4 ± 1.1 (14)	4.1 ± 0.1 (14)	−68.4 ± 0.7 (10)	5.2 ± 0.4 (10)
Δ (1 mM compared to 4 mM)	0	0	0	0	+1.1	+0.2
**A204E**						
4 mM K^+^	−35.0 ± 1.1 (28)	6.4 ± 0.2 (28)	−76.0 ± 0.7 (28)	3.7 ± 0.1 (28)	−77.0 ± 0.8 (14)	5.1 ± 0.2 (14)
1 mM K^+^	−27.4 ± 1.4 (18)	7.9 ± 0.2 (18)	−72.9 ± 0.9 (18)	4.1 ± 0.1 (18)	−74.8 ± 0.6 (9)	4.1 ± 0.2 (9)
Δ (1 mM compared to 4 mM)	+7.6***	+1.5**	+3.1*	+0.4**	+2.2	−1.0**
**A204E values compared to WT values**						
4 mM K^+^	−3*	2.5***	−6.2***	−0.3***	−7.5**	0.1
1 mM K^+^	+4.5*	3.9***	−3.5*	0	−6.4***	−1.1*

V_1/2_, midpoint for activation or inactivation (mV); k, slope factor for activation or inactivation. The numbers of cells (n) are in brackets. Values are means ± sem. *P < 0.05; **P < 0.01; ***P < 0.001. Δ, variation of V_1/2_ and k for WT (1 mM compared to 4 mM K^+^_ext_), A204E (1 mM compared to 4 mM K^+^_ext_) or A204E compared to WT at 1 and 4 mM K^+^_ext_.

## Discussion

We recently reported a Caucasian patient suffering from PP combining symptoms of hyperPP to hypokalaemic episodes of paralysis who was heterozygous for the p.R1451L missense mutation in the Na_v_1.4 voltage sensor segment of domain IV (DIVS4)^11^. An unrelated Chinese patient was subsequently reported to present this phenotype, who was homozygous for the same (p.R1451L) mutation in Na_v_1.4 whereas heterozygous individuals for the mutation in this family were asymptomatic or displayed electrical myotonia^20^. Here, we report a third patient with the unusual combination of hyper- and hypo-PP symptoms who was heterozygous for a novel Na_v_1.4 mutation located in DIS3. Our data confirm the relationship between this newly described phenotype of PP and Na_v_1.4 dysfunction and give critical elements to understand its pathophysiology, especially its hypoPP component.

The patient suffered from two types of paralytic episodes since his teenage. Some episodes occurred several times in the day and lasted less than one hour, whereas others were longer and were concomitant to hypokalaemia. Muscle stiffness in cold environment and myotonic discharges at EMG argue for the diagnosis of hyperPP since clinical overlap between myotonia and hyperPP is frequent, and CMAP responses to short and long exercise (Mc Manis) tests agree with this diagnosis. However, hyperkalaemia was never documented during attacks of paralysis by contrast to hypokalaemia. Furthermore, we did not find clinical signs for another physiological dysfunction nor identify another genetic cause (mutation in *KCNJ2* or *CACN1AS*) that may account for the hypokalaemic episodes of paralysis^11^. Transient hypokalaemia is a possible event at the end of paralytic episodes in hyperPP as a consequence of K^+^ muscle reuptake and renal elimination^21^. We exclude this possibility as the cause of hypokalaemia documented during paralytic episodes in the patient reported here since he suffered from matinal paralytic episodes following carbohydrate-rich diners as usually observed in hypoPP. Moreover, an episode of hypoPP was undoubtedly induced by carbohydrate rich-food when the patient was under clinical supervision.

The p.A204E mutation reported here for the first time, is located in the DIS3 region of Na_v_1.4, which is a region of Na_v_1.4 poor in disease-related mutations. The biophysical changes induced by p.A204E on Na_v_1.4 include significant hyperpolarized shift of the voltage-dependence of steady state activation and fast inactivation and accelerated entry into fast inactivation. These changes are similar to those resulting from gain-of-function mutations causing hyperPP such as p.Ile1495Phe located in DIVS4 of Na_v_1.4, and may well account for this clinical component reported here^22^. The interpretation of the hypokalaemic paralytic events in regard to the biophysical changes of Na_v_1.4 induced by p.A204E in heterologous cells is more challenging. Indeed, we did not detect any gating pore current in *Xenopus* oocytes expressing A204E mutant channels. The hypokalaemic paralytic episodes linked to p.A204E are then physiologically distinct from those observed in familial hypoPP, which result from a depolarizing gating pore current induced by the substitution of one positively-charged residue of voltage-sensor S4 segments. Nevertheless, the comparison between the biophysical changes induced by p.A204E to those induced by p.R1451L — that substitutes one positively-charged residue in DIVS4 but does not induce gating pore current^11,20^— gave clues for understanding the occurrence of this novel phenotype (Table 3). The p.A204E and p.R1451L mutations share the absence of gating pore current, gain-of-function effects on Na_v_1.4 biophysical properties such as increased window current, and loss-of-function effects including 2-fold reduction in current density, lower steepness of activation curve, accelerated entry into fast inactivation (at −50 mV), and slower recovery from slow inactivation. These similarities support the hypothesis that Na_v_1.4 loss-of-function may favor paralytic attacks^23^. Indeed, Na_v_1.4 mutations in S4 segments linked to the familial form of hypoPP have been reported to exert loss-of-function effects with reduced current density and enhanced fast or slow inactivation in addition to promote gating pore current^24–27^. However, two-fold reduction in skeletal muscle Na_v_1.4 current in *Scn4a* haploinsufficient mice (i.e. heterozygous for a null *Scn4a* allele) induced muscle weakness in response to repetitive stimulations but not in response to PP triggering factors (K^+^, glucose and insulin challenges), thereby clinically mimicking myasthenia *i.e*. fatigable muscle weakness in response to repetitive stimulation rather than PP^28^. P.A204E should then have impacts favoring hypoPP in addition to its loss-of-function effects. Interestingly, we showed that lowering K^+^_ext_ to 1 mM strengthens the loss-of-function effects produced by p.A204E on Na_v_1.4 in functional assays whereas it did not impact the WT channel. The most drastic effect was on steady-state activation since lowering K^+^_ext_ from 4 to 1 mM shifted its voltage-dependence from hyperpolarization to depolarization compared to WT channels, and further lowered the steepness of the activation curve. K^+^ sensitivity of Na^+^ channel myotonia due to dominant *SCN4A* mutations is well known^1,4^. [K^+^]_ext_ modified the biophysics of p.G1306A/E/V channels — missense mutations located in the cytoplasmic DIII-DIV linker that result in K^+^-aggravated myotonia — in heterologous cell expression system^19,29^. The location of the A204 residue near the extracellular side of DIS3, and the negatively charged Glu residue could in part account for the K^+^ sensitivity induced by p.A204E, although an indirect mechanism through changes in K^+^ channels activity cannot be excluded.

**Table 3:** Comparison of biophysical parameters of A204E and R1451L mutant channels recorded at 4 mM K^+^_ext_.

Mutation	Current density	Steady state activation		Fast inactivation			Window current^$^	Slow inactivation
		V_1/2_	k_v_	V_1/2_	k_v_	τ		
A204E	50%	−3 mV	164%	−6.3 mV	92%	28%	7-fold increase	Faster entry, slower recovery
R1451L	46%	*ns*	136%	−19.6 mV	224%	30%	7-fold increase	Slower recovery

The parameters considered here are those reported to be statistically different between the mutant (A204E or R1451L) and the WT channels (this study for A204E or Poulain *et al*.^11^ for R1451L). All data are expressed as percent of WT values (equal to 100%) except the midpoints for steady-state activation and fast inactivation (V_1/2_). V_1/2_, differences (mV) between the midpoints for activation or fast inactivation of the mutant channels (A204E or R1451L) and those of WT channels (WT); k, slope factor for voltage dependence of activation or inactivation; τ, time constants of entry into fast inactivation at −50 mV. ^$^Probability to be in the window current. *ns*, not significant.

Substituting the neutral Ala204 residue by the negatively-charged Glu residue probably has several structural consequences on Na_v_1.4. First, the helix-forming propensity of Glu is lower than Ala, which may destabilize the DIS3 segment. Second, adding one negatively-charged residue in DIS3, which contains only two negatively-charged residues (Asp197 and Glu208), may perturb the upward motion of the DIS4 gating charges through their electrostatic interactions with acidic Glu161 and Glu171 (located in DIS2) and Asp197 (located in DIS3) amino acids within the voltage-sensor domain^3,30,31^. Using molecular dynamic simulations based on the sequence of the bacterial sodium channel NaVAb, the substitution of the hydrophobic Ile1455 — located between the positively-charged Arg1451 and Arg1454 gating charge residues in DIVS4 — by the polar Thr residue was proposed to hinder the upward mobility of DIVS4^32^. That illustrates the fact that substituting an amino acid residue in voltage-sensor domain other than one positively-charged one may impact S4 motion. With the emergence of next-generation sequencing technologies to identify causative mutations for clinical purpose, interpreting the functional significance of novel variants using computational tools is a growing need. The recent 3.2 Å resolution of human Na_v_1.4 structure by cryo-electron microscopy and the structural mapping of known disease mutations will pave the way to best predict the molecular impact of any new amino acid variant on Na_v_1.4 kinetics^31^.

Among the 70 disease-causing variations reported in Na_v_1.4, only one is located in DIS3 (p.Met203Lys (p.M203K))^33,34^. Precisely, p.M203K results in recessively-inherited congenital myopathy and exerts a loss-of-function effect by decreasing Na^+^ current density and by shifting the voltage-dependence of activation toward depolarized potentials^34^. P.A204E is located next to p.M203K, and also exerts a loss-of-function effect on Na_v_1.4. Why p.A204E is associated to a dominantly-inherited phenotype whereas p.M203K is recessively-inherited and cause congenital myopathy may be explained by dosage effect and voltage-gated Na^+^ channels dimerization^35^. In congenital myopathy, p.M203K was found to be heteroallelic to the p.Tyr1593X nonsense mutation, which is predicted to truncate the intracellular C-terminal tail of Na_v_1.4. The dimer forms by the interaction of the truncated p.Tyr1593X channel with p.M203K channel probably leads to a complex not addressed at the membrane, further reducing the amount of functional Na_v_1.4 channels. Another and not exclusive explanation lies on the well-known influence exerted by genetic and environmental factors on the clinical expressivity of any disease-causing mutation that remain to be identified for muscle sodium channelopathies with the help of models more physiologic than heterologous cells^36^.

Altogether, our data stress the existence of a clinical form of PP due to dominant Na_v_1.4 mutations that combines characteristics of hyperPP to hypokalemic episodes of paralysis. The hyperPP component would result from gain-of-function effects of the mutation whereas the hypokalaemic episodes would result from the exacerbation of loss-of-function effects on Na_v_1.4 by hypokalaemia whose physiological basis remains to be determined. Our present work strengthens the role played by the loss-of-function effects of S4 mutations on Na_v_1.4 current in familial hypoPP.

## Materials and Methods

### Genetic analyses

Genomic DNA was extracted from blood leukocytes and was used to amplify exons and flanking introns of *SCN4A* and *CLCN1* by PCR. The amplicons were sequenced using the ABI Dye terminator chemistry and an ABI DNA sequencer (ABI 3100; Applied Biosystems, Foster City, CA, USA). Capture-Next Generation Sequencing experiment was performed to exclude any mutation in *CACNA1S* and *KCNJ2*.

### *In vitro* electrophysiology in HEK293 cells

The c.611 C >  A mutation was introduced by site-directed mutagenesis into the cDNA encoding the human Na_v_1.4 isoform that was cloned into the pRc/CMV vector (Quick change lightning Site-Directed Mutagenesis kit; Agilent Technologies). Sanger sequencing of the full cDNA was performed after directed mutagenesis to confirm the C to A transversion and exclude the occurrence of any unexpected mutation. We used the same methodology as the one employed in a previous study for transient transfection of human embryonic kidney (HEK) 293 T cells using the calcium phosphate method with some modifications^37^. Briefly, Plasmid DNAs encoding wild-type (WT) or mutant h Na_v_1.4 α subunit, the human Na^+^ channel β1 subunit (fourfold molar excess over α subunit DNA), and a CD8 marker were cotransfected by the calcium phosphate method. At 2 days after transfection, the HEK cells were trypsinized briefly and passaged for electrophysiological recording. Individual transfection-positive cells were identified by labeling with anti-CD8 antibody cross-linked to microbeads (Dynal, Great Neck, NY)^38^.

Na^+^ currents were recorded at room temperature (≈22 °C) using the whole-cell configuration of the patch clamp techniques with an Axopatch 200B amplifier (Molecular Devices). Data were low-pass filtered with an eight-pole Bessel filter at 2 kHz and acquired at 50 kHz with pClamp10.2 software (Molecular Device). Cells with peak currents <1 or>5 nA upon step depolarization from −120 mV to −10 mV were excluded from analyses. The pipette internal solution contained 105 mM CsF, 35 mM NaCl, 10 mM ethylene glycol tetra-acetic acid (EGTA), and 10 mM Cs-HEPES (pH 7.4). The bath solution contained 140 mM NaCl, 4 mM KCl, 2 mM CaCl_2_, 1 mM MgCl_2_, 5 mM glucose, and 10 mM Na-HEPES (pH 7.4). A bath solution with a low (1 mM) K^+^ concentration was used to determine the K^+^ sensitivity of the WT and A204E channels. The osmolarity of the extracellular solution was then adjusted to 280 mOsm with glucose. All chemicals were purchased from Sigma-Aldrich^®^. Data were analyzed with Clampfit (Molecular Device). Curve-fitting was performed using Origin^®^ (OriginLab^®^).

Na^+^ conductance was calculated from the peak current I_Na_ using the equation 1:

1 $${{\rm{G}}}_{{\rm{Na}}}={{\rm{I}}}_{{\rm{Na}}}/({\rm{V}}-{{\rm{E}}}_{{\rm{Na}}})$$

where V is the test potential and E_Na_ is the reversal potential. Voltage-dependence of activation was quantified by fitting Na^+^ conductance to voltage using the Boltzmann equation 2:

2 $$G={G}_{{\rm{\max }}}/[1+\exp \,(-\,(V-{V}_{1/2})/k)]$$

where *V*_1/2_ is the half-maximum voltage and *k* is the slope factor. Steady-state fast inactivation was fitted to the Boltzmann equation 3:

3 $$I/{I}_{{\rm{\max }}}=1/[1+exp\,((V-{V}_{1/2})/k)]$$

where I is the current, V_1/2_ is the voltage at which the channels are half-maximally inactivated, and k is the slope factor.

The probability of being within the window current was calculated through the equation 4:

4 $$(1/(1+\exp \,(({{\rm{V}}}_{1/2{\rm{act}}}-{\rm{V}})/{{\rm{k}}}_{{\rm{act}}}))\,\times \,(1+\exp \,(({\rm{V}}-{{\rm{V}}}_{1/2{\rm{inact}}})/{{\rm{k}}}_{{\rm{inact}}})))$$

The time courses of entry into fast inactivation and recovery from fast and slow inactivation were evaluated by using two-pulse protocols described in the figures legends^37^. Recovery curves were fitted to and the time constants were calculated from the single exponential equation 5:

5 $${\rm{y}}={{\rm{y}}}_{{\rm{o}}}+{\rm{Aexp}}\,(-\,x/{\boldsymbol{\tau }})$$

where y_o_ is the asymptote of the fit, A is the maximal amplitude, τ is the time constant and x is time. Statistical significance was determined using the Student’s unpaired *t* test with *P* < 0.05 considered as significant.

### Gating pore measurements

We used the same methodology as the ones employed in previous studies for oocyte handling, RNA injection, and current recordings^39,40^. Oocytes were surgically removed from frogs anesthetized with MS-222 (Sigma-Aldrich^®^). The oocytes were treated with 2 mg/mL collagenase (collagenase type 1 A from *Clostridium histolyticum*, Sigma-Aldrich^®^) in oocyte recipe OR2 solution for 2 h and were incubated for at least 1 h at 18 °C in OR3 solution before RNA injection. Stage IV and V oocytes were microinjected with mRNA corresponding to the cDNA of the rat *Scn4a* gene and the β1 regulatory subunit and placed at 18 °C in OR3 solution until voltage-clamp recording experiments. The OR2 solution was composed of 82.5 mM NaCl, 2.5 mM KCl, 1 mM MgCl2, and 5 mM HEPES. The pH was adjusted to 7.6 at room temperature using 1 M NaOH. The OR3 solution was composed of a 1:2 dilution of Leibovitz’s L-15 medium supplemented with 15 mM HEPES and 50 mg/mL of gentamycin. The pH was adjusted to 7.6 at room temperature using 1 M NaOH for the two ORs.

A Q5^®^ site-directed mutagenesis kit (New England Biolabs® Inc) was used to insert the A204E substitution in the rat *Scn4a* cDNA placed in pPol *Xenopus laevis* oocyte expression vector^40^. The substitution equivalent to p.R222G in human Na_v_1.4 was used as a positive control for gating pore current^10^. Sanger sequencing of the full cDNA was performed to confirm site-directed mutagenesis and exclude any unexpected mutation. The rat *Scn4a* and its β_1_ subunit mRNAs were transcribed using the HiScribe T7 Quick kit (New England Biolabs^®^ Inc) and were co-injected in oocytes in a 1:1 mass ratio.

The oocytes were impaled with < 1MΩ electrodes filled with 3 M KCl and were voltage-clamped with an OC-725C Oocyte Clamp (Warner Instruments Corp.). The currents were filtered at 5 kHz (−3 dB; four-pole Bessel filter) and were collected using pClamp (Molecular Devices) and analyzed using Clampfit (Molecular Devices). Bath solution contained 60 mM Na^+^-methanesulfonate with 60 mM guanidine sulfate, 1.8 mM CaSO4 and 10 mM HEPES (pH 7.4). All chemicals were purchased from Sigma-Aldrich^®^, except for Leibovitz’s L-15 medium, which was purchased from ThermoFisher Scientific. All experiments were carried out at room temperature (≈22 °C).

### Standard protocol approvals, registrations, and patient consents

The study was approved by a French Ethical Review Board according to national relevant guidelines and regulations (DC-2012-1535 and AC-2012-1536). The patient gave written informed consent for blood specimen, genetic testing and research. All experimental procedures involving *Xenopus laevis* oocytes were approved by the Institutional Animal Care Committee of Laval University and were in compliance with the principles and guidelines of the Canadian Council on Animal Care (Approval 2011155-1).

## Data Availability

The anonymized data that support the findings of this study are available from the corresponding authors upon reasonable request.
